# Tolerance of the freeze-dried mouse sperm nucleus to temperatures ranging from −196 °C to 150 °C

**DOI:** 10.1038/s41598-019-42062-8

**Published:** 2019-04-05

**Authors:** Sayaka Wakayama, Daiyu Ito, Yuko Kamada, Shigenobu Yonemura, Masatoshi Ooga, Satoshi Kishigami, Teruhiko Wakayama

**Affiliations:** 1 Advanced Biotechnology Center, University of Yamanashi, 4-4-37 Takeda, Kofu-shi, Yamanashi, Japan; 2 Faculty of Life and Environmental Sciences, University of Yamanashi, Yamanashi, Japan; 3 RIKEN Center for Biosystems Dynamics Research, Kobe, 650-0047 Japan; 4 Department of Cell Biology, Tokushima University Graduate School of Medical Sciences, Tokushima, 770-8503 Japan

## Abstract

It has long been believed that tolerance against extreme environments is possible only for ‘lower’ groups, such as archaea, bacteria or tardigrades, and not for more ‘advanced’ species. Here, we demonstrated that the mammalian sperm nucleus also exhibited strong tolerance to cold and hot temperatures. When mouse spermatozoa were freeze-dried (FD), similar to the anhydrobiosis of Tardigrades, all spermatozoa were ostensibly dead after rehydration. However, offspring were obtained from recovered FD sperm nuclei, even after repeated treatment with conditions from liquid nitrogen to room temperature. Conversely, when FD spermatozoa were heated at 95 °C, although the birth rate was decreased with increasing duration of the treatment, offspring were obtained even for FD spermatozoa that had been heat-treated for 2 h. This period was improved up to 6 h when glucose was replaced with trehalose in the freeze-drying medium, and the resistance temperature was extended up to 150 °C for short periods of treatment. Randomly selected offspring grew into healthy adults. Our results suggest that, when considering the sperm nucleus/DNA as the material that is used as a blueprint of life, rather than cell viability, a significant tolerance to extreme temperatures is present even in ‘higher’ species, such as mammals.

## Introduction

It is well known that tolerance to extreme environments is observed not only in archaea and bacteria but also in tardigrades and the larvae of Chironomids^1^. When tardigrades become immobile and shrink into the form that is known as the ‘tun’ or ‘anhydrobiotic’ state^2^, they acquire a strong tolerance to high or low temperatures and to lethal levels of radiation; moreover, some of them survive even when exposed to outer space^3–5^. Conversely, it has long been believed that ‘higher’ species, such as mammals, cannot enter an anhydrobiotic state, and none of these species survived when exposed to such extreme conditions^6^. However, previously, we and others have demonstrated that the dead bodies or frozen cadavers of mammalian species could be ‘revived’ when their spermatozoa or somatic cell nuclei were transplanted into oocytes to produce fertilized or cloned offspring^7–10^. In addition, recently, we and others have demonstrated that the complete drying of mammalian spermatozoa—similar to the anhydrobiotic state of Tardigrades—allowed their preservation even at room temperature without losing their potential for supporting development after fertilization^11–15^. These findings suggest that, although the entire mammalian body is susceptible to extreme environments, its cell nuclei can retain vital potential and that healthy offspring can be generated from them using recent reproductive biotechnology. If such nuclei exhibit a strong tolerance against extreme environments for long periods, it might be possible to resurrect extinct species from frozen cadavers^7,8^, rescue endangered species from waste products^9,16,17^, preserve genetically modified mouse strains at room temperature^15^ and conserve genetic resources against disasters on Earth (such as the loss of electric power) using storage on the Earth’s moon^18^. Recently, several reports have suggested that the environmental tolerance of DNA from freeze-dried (FD) spermatozoa is increased compared with that of fresh cells; however, it has not been confirmed whether animals could be generated from such spermatozoa^19,20^ and the limitations of this technology are not clear. From this perspective, it will be interesting to determine whether the mammalian nucleus/DNA, rather than cells, has a strong tolerance to extreme environments.

For these reasons, in this study, we attempted to generate offspring from spermatozoa treated with low temperature, frequent temperature changes or high temperature and determined the limits of tolerance of the sperm nucleus after freeze-drying.

## Results

### Resistance against frequent temperature changes

**Figure 1:**
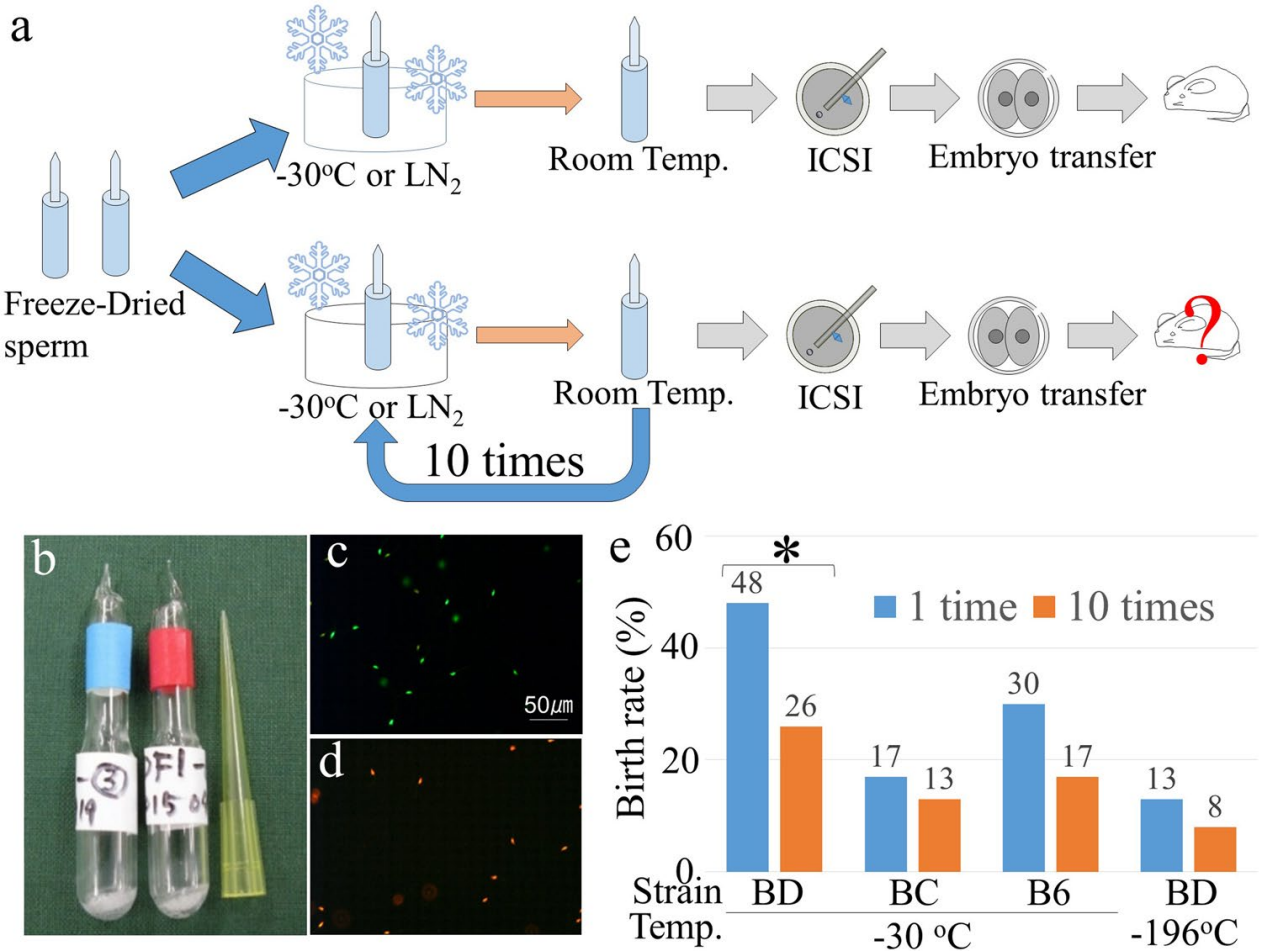
The tolerance of FD spermatozoa to frequent temperature changes enabled full-term development after ICSI. (**a**) Schematic diagram of the repeated cooling of FD spermatozoa from room temperature to −30 °C or LN_2_, and generation of offspring. ICSI, intracytoplasmic sperm injection. (**b**) Ampoules of FD spermatozoa. (**c**,**d**) Live/dead staining of FD spermatozoa before (**c**) and after (**d**) freeze-drying using live/dead assay kits. Green spermatozoa were classified as being alive and red spermatozoa were classified as being dead. (**e**) The production rate of offspring using repeatedly cooled FD spermatozoa. FD spermatozoa were cooled one or 10 times. The mouse strain sources for the spermatozoa were: BD, BDF1; BC, BCF1; and B6, C57BL/6N.

To assess the tolerance of FD spermatozoa to temperature, first, FD spermatozoa were treated with temperatures ranging from −30 °C or −196 °C (liquid nitrogen, LN_2_), to RT (around 25 °C) and up to 10 times RT (Fig. 1a). The examination of FD spermatozoa immediately after freeze-drying treatment revealed that, although none of them survived after rehydration (Fig. 1b–d; Supplemental Table 1), healthy offspring could be obtained after their microinjection into fresh oocytes. Treatment with frequent temperature changes did not alter the morphology of rehydrated FD spermatozoa, even when these temperature changes were repeated 10 times. When such spermatozoa were microinjected into fresh oocytes, most of them fertilized normally (Table 1). Healthy offspring were obtained after embryo transfer into recipient females, although the success rates of the production of live offspring were decreased compared with controls (Fig. 1e). Similar results were obtained for the B6C3F1 and C57BL/6 mouse strains (Table 1). Moreover, healthy offspring were obtained even when temperature changes from −196 °C to RT were repeated 10 times (Table 1). Randomly selected offspring were fostered and their growth to adulthood was verified. Thus, the nuclei of FD mouse spermatozoa exhibited a strong tolerance to frequent temperature changes between RT and −30 °C or even −196 °C.

**Table 1:** Full term development of mouse oocyte injected with freeze-dried spermatozoa treated with −30 °C or LN2 repeatedly up to ten times.

Mouse strain*	Treated temp.	No. repeating	No. injected oocytes	No. (%) of activated oocytes			No. (%) of embryos developed to 2-cell** [recipient]	No. (%) [min-max] of offspring
				2PN	1PN	0PN		
BDF1	−30 °C	1	35	27 (77)	8 (22)	0	27 (77) [1]	13 (48) [13]^a^
		10	48	43 (89)	3 (6)	2 (4)	42 (87) [2]	11 (26) [4, 7]^b^
BCF1	−30 °C	1	26	14 (54)	1 (4)	11 (42)	13 (50) [1]	4 (30) [4]
		10	40	29 (72)	1(2)	10 (25)	23 (57) [1]	4 (17) [4]
B6	−30 °C	1	45	42 (93)	1 (2)	2 (3)	30 (65) [2]	4 (13) [1, 3]
		10	50	37 (74)	13 (35)	0	34 (68) [2]	3 (8) [0, 3]
BDF1	−196 °C (LN_2_)	1	73	64 (87)	8 (11)	1 (1)	62 (96) [3]	11 (17) [0–6]
		10	66	53 (80)	7 (10)	6 (9)	52 (78) [3]	7 (13) [0–5]

^a^vs. ^b^P < 0.05. Other were no significant difference.

PN: pseudo-pronucleus.

*BDF1: C57BL/6N × DBA/2; BCF1: C57BL/6N × C3H/He; B6: C57BL/6N.

**All embryos were transferred into oviduct of recipient female.

### Tolerance to 95 °C

**Figure 2:**
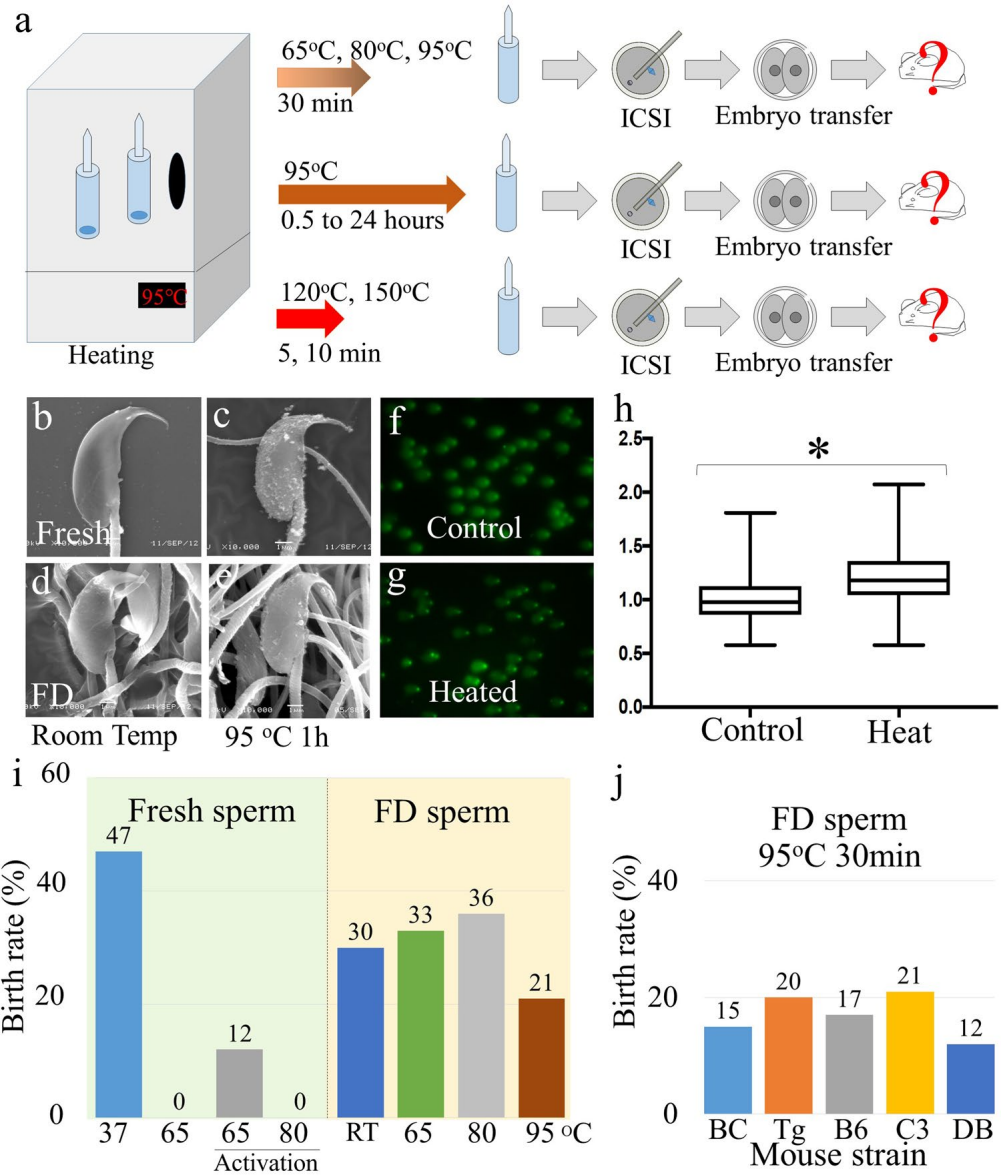
The tolerance of FD spermatozoa to heat-treatment enabled full-term development after ICSI. (**a**) Schematic diagram of the heat-treated FD spermatozoa and generation of offspring. FD sperm ampoules were heated in an oven in three different ways. First (upper): at 65 °C, 80 °C or 95 °C for 30 min; second (middle): at 95 °C for 30 min to 24 h; and third (bottom): at 120 °C or 150 °C for 3–20 min. (**b–e**) Morphology of FD spermatozoa, as assessed using scanning electron microscopy. (**b**,**c**) Fresh spermatozoa. (**d**,**e**) FD spermatozoa. (**b**,**d**) Without heat-treatment. (**c**,**e**) Treated at 95 °C for 1 h. (**f–h**) Comet DNA breakage assays of heat-treated FD spermatozoa. (**f**,**g**) Comet tail of FD spermatozoa without heat-treatment (**f**) and with heat-treatment at 95 °C for 30 min (**g**). (**h**) Comparison of the comet tail lengths of FD spermatozoa stored in control ampoules or in those treated at 95 °C for 30 min. The lengths of comet tails were standardized against the mean lengths of control spermatozoa. Asterisk denotes statistically significant differences between samples (*P* < 0.05). (**i**) The production rate of offspring using fresh and FD spermatozoa that were heat-treated for 30 min. In fresh spermatozoa, oocytes were activated artificially after the injection of spermatozoa that were treated at a temperature ≥65 °C. (**j**) The production rate of offspring using FD spermatozoa that were heat-treated at 95 °C for 30 min. The mouse strain sources for the spermatozoa were: BC, B6C3F1; Tg, 129B6F1-Tg; B6, C57BL/6N; C3, C3H/He; and DB, DBA/2.

Next, we examined whether FD mouse spermatozoa would show tolerance to a high temperature (Fig. 2a) because most tardigrade species show a rapid decline in survival when they are exposed to temperatures >90 °C^21^. When fresh spermatozoa were exposed to a temperature >80 °C for 30 min, the surfaces of most heated spermatozoa became ragged with many small granules (Fig. 2b,c; Supplemental Table 2), and no offspring were obtained even when oocytes were activated artificially (Fig. 2i; Table 2), as reported previously^22,23^. Conversely, treatment of FD spermatozoa with temperatures up to 95 °C for 30 min did not alter the morphology of rehydrated spermatozoa compared with fresh spermatozoa (Fig. 2d,e; Supplemental Table 2). The level of DNA damage in heated FD sperm nuclei increased significantly compared with non-heated FD spermatozoa (Fig. 2f–h; Supplemental Table 3). When heat-treated FD spermatozoa for 65 °C, 80 °C and 90 °C for 30 min were injected into fresh oocytes, although the overall rate of production of offspring was slightly decreased, healthy offspring were still obtained (Fig. 2i, Table 2). Randomly selected offspring were fostered and their growth to adulthood was verified. This heat tolerance was confirmed for the B6C3F1, 129B6F1-Tg, C57BL/6, C3H/He and DBA/2 mouse strains (Fig. 2j, Table 3).

**Table 2:** Full term development of mouse oocyte injected with freeze-dried spermatozoa after several temperature treatment for 30 minuets.

Sperm condition	Oocyte activation	Heat temp. (°C)	No. injected oocytes	No. (%) of activated oocytes			No. (%) of embryos developed to 2-cell* [recipient]	No. (%) [min-max] of offspring
				2PN	1PN	0PN		
Fresh sperm	−	No heat	53	53 (100)	0	0	53 (100) [3]	25 (47) [7–10]
		65	63	0	0	63 (100)	5 (8) [1]	0
		>80	81	0	0	81 (100)	3(4) [1]	0
Fresh sperm	+	65	99	56 (56)	43 (43)	0	68 (68) [4]	6 (12) [1–2]
		80	38	10 (26)	28 (73)	0	21 (55) [1]	0
		95	29	0	29 (100)	0	21 (72) [1]	0
FD sperm	−	No	34	33 (97)	1 (3)	0	26 (76) [1]	8 (30) [8]
		65	35	34 (97)	1(3)	0	18 (51) [1]	6 (33) [6]
		80	63	60 (95)	3 (5)	0	60 (95) [3]	22 (36) [5–10]
		95	59	50 (85)	9 (15)	0	19 (32) [2]	4 (21) [1, 3]

There were no significant difference between treated temperature of FD spermatozoa.

PN: pseudo-pronucleus.

*All embryos were transferred into oviduct of recipient female.

**Table 3:** Full term development of mouse oocyte injected with freeze-dried spermatozoa of different mouse strains after 95 °C treatment for 30 minutes.

Mouse Strain*	No. injected oocytes	No. (%) of activated oocytes 6 h after ICSI			No. (%) of embryos developed to 2-cell** [recipient]	No. (%) [min-max] of offspring
		2PN	1PN	0PN		
BCF1	48	30 (62)	0	18 (37)	20 (41) [2]	3 (15) [0, 3]
129B6F1Tg	28	10 (36)	2 (7)	16 (57)	10 (36) [1]	2(20) [2]
B6	45	29 (64)	3(6)	13 (29)	23 (51) [2]	4(17) [0, 4]
C3H	54	29 (54)	8 (14)	17 (31)	28 (52) [2]	6(21) [3, 3]
DBA2	42	37 (88)	5 (12)	0	33 (78) [2]	4(12) [1, 3]

PN: pseudo-pronucleus.

*BCF1: C57BL/6N × C3H/He; 129B6F1Tg: 129/Sv-GFPTg × C57BL/6N-GFPTg: B6: C57BL/6N.

**All embryos were transferred into oviduct of recipient female.

**Figure 3:**
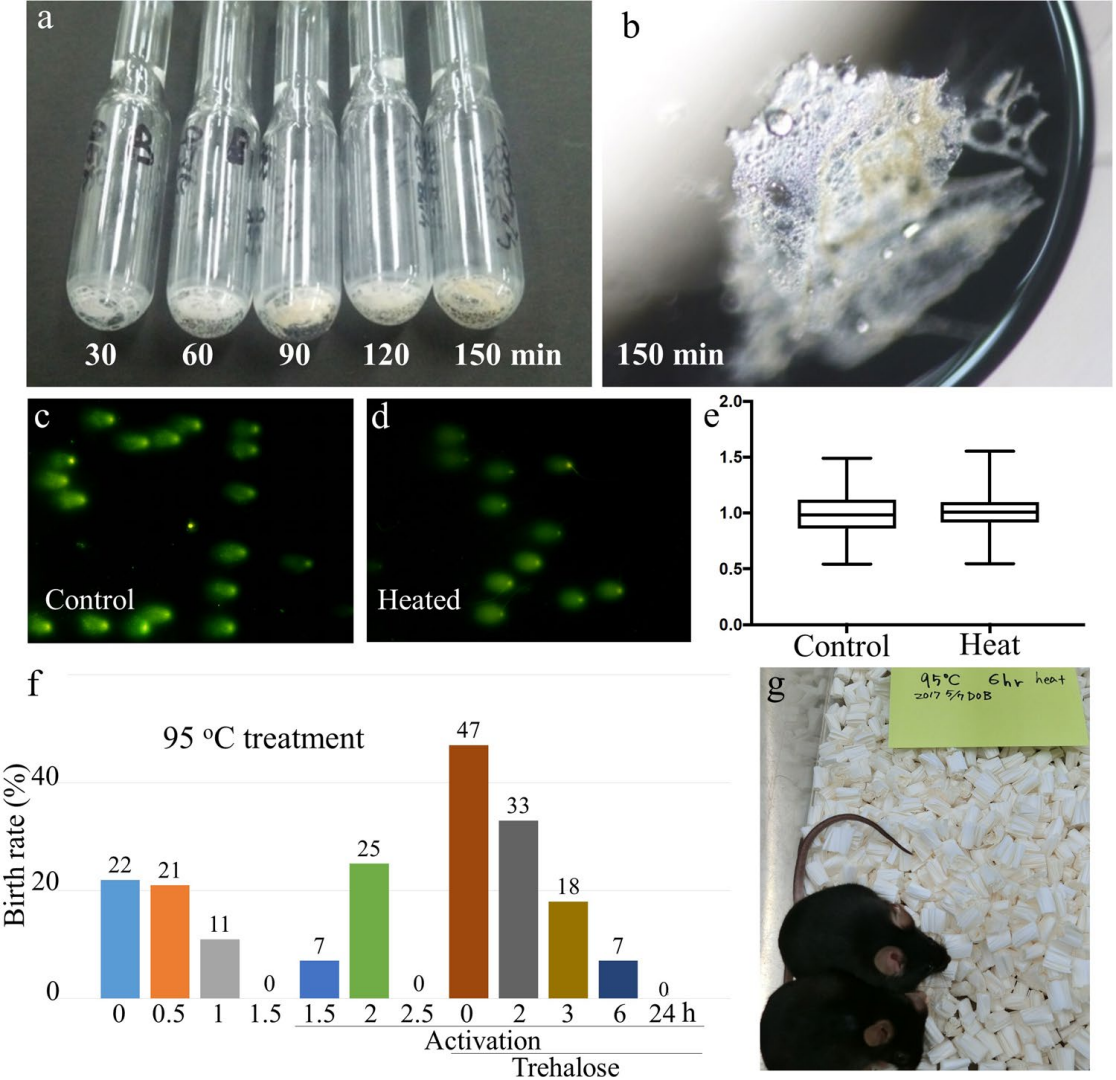
The maximum potential of heat-treated FD spermatozoa for enabling full-term development after ICSI. (**a**,**b**) Scorched ampoules. Ampoules that were heated at 95 °C for more than 2 h were scorched and no spermatozoa were collected after rehydration. (**c–e**) Comet DNA breakage assays of heat-treated FD spermatozoa. (**c**,**d**) Comet tail of FD spermatozoa, which were freeze-dried in medium containing trehalose instead of glucose. (**c**) Without heat-treatment. (**d**) With heat-treatment at 95 °C for 30 min. (**e**) Comparison of the comet tail lengths of FD spermatozoa stored in control ampoules or in those that were treated at 95 °C for 30 min. The medium contained trehalose instead of glucose. The lengths of comet tails were standardized against the mean lengths of control spermatozoa. (**f**) The production rate of offspring using FD spermatozoa that were treated at 95 °C for up to 24 h. In the latter experiments, oocytes were activated artificially. In addition, some ampoules were freeze-dried in medium containing trehalose instead of glucose. (**g**) Offspring derived from FD spermatozoa that were treated at 95 °C for 6 h. All examined offspring grew to adulthood.

We then examined the duration of the tolerance of the nuclei of FD spermatozoa to 95 °C treatment. In this experiment, some oocytes were activated artificially just after sperm microinjection because, even for FD spermatozoa, the oocyte activation capacity was decreased with increased heat-treatment duration. In addition, as the ampoules of FD spermatozoa became visibly scorched after 2 h of heat-treatment, because of the Maillard reaction of glucose (Fig. 3a,b), we replaced glucose with trehalose in the medium in later experiments. Trehalose is a cryoprotectant that is used for cell freezing or drying^24,25^, and many tardigrade species accumulate it to prepare for exposure to such extreme environments^26^. When glucose was replaced with trehalose, the level of DNA damage in FD sperm nuclei that were heated at 95 °C for 30 min was similar to that of non-heated FD spermatozoa (Fig. 3c–e; Supplemental Table 3). Using those FD spermatozoa, live and healthy offspring were obtained even from the FD spermatozoa that were exposed to a temperature of 95 °C for 6 h (Fig. 3f,g; Table 4). Randomly selected offspring were fostered and their growth to adulthood was verified (Fig. 3g). A similar, but lower, tolerance was also observed for spermatozoa of the C57BL/6 mouse strain (Supplemental Table 4).

**Table 4:** Full term development of mouse oocyte injected with freeze-dried spermatozoa treated with 95 °C for up to 24 h with or without oocyte activation and trehalose.

Period of 95 °C treated	Oocyte activation	Trehalose	No. injected oocytes	No. survived	No. (%) of activated oocytes 6 h after ICSI		No. (%) of embryos developing to 2-cell *	No. (%) [min-max] of offspring
					2PN	1PN		
0	−	−	30	28	27 (96)	1 (4)	23 (85) [2]	5 (22) [0, 5]
0.5 h	−	−	67	59	50 (86)	9 (14)	19 (32) [2]	4 (21) [0, 4]
1 h	−	−	48	37	32 (86)	5 (14)	26 (70) [1]	3 (11) [3]
1.5 h	−	−	70	40	14 (35)	26 (65)	8 (2) [1]	0
1.5 h	+	−	105	90	68 (75)	18(25)	56 (62) [2]	4 (7) [0, 4]
2 h	+	−	60	58	52 (89)	6(11)	52 (89) [2]	13 (25) [5, 8]
2.5 h	+	−	Scorched	−				
0	+	+	60	53	53 (100)	0	53 (100) [3]	25 (47) [3–13]
2 h	+	+	50	36	35 (97)	1 (3)	33 (91) [2]	11 (33) [4, 7]
3 h	+	+	80	66	56 (84)	10 (16)	54 (81) [3]	10 (18) [0–6]
6 h	+	+	75	65	53 (81)	12 (19)	52 (80) [3]	4 (7) [0–3]
24 h	+	+	130	113	61 (53)	52 (47)	51 (45) [3]	0

PN: pseudo-pronucleus.

*All embryos were transferred into oviduct of recipient female.

### Tolerance to a temperature >100 °C for short periods

We also examined the tolerance of FD spermatozoa against temperatures of more than 100 °C. When FD spermatozoa were treated at 120 °C and 150 °C, the maximum tolerance periods were 10 min and 3 min, respectively (Table 5), and healthy offspring were obtained from such spermatozoa after microinjection. Randomly selected offspring were fostered and their growth to adulthood was verified. These results suggest that the FD mouse sperm nucleus is tolerant to temperatures >100 °C for short periods, which is better than the tolerance shown by some tardigrade species^21^.

**Table 5:** Full term development of mouse oocyte injected with freeze-dried spermatozoa treated with 120 °C or 150 °C for short periods with our without trehalose.

Heat temp	Trehalose	Heating period	No. injected oocytes	No. survived	No. (%) of activated oocytes 6 h after ICSI		No. (%) of embryos developing to 2-cell*	No. (%) [min-max] of offspring
					2PN	1PN		
120 °C	−	3 min	100	85	77 (91)	8 (9)	75 (88) [3]	5 (7) [0–3]
		5 min	160	130	122 (94)	8 (6)	111 (85) [4]	0
150 °C	−	3 min	100	80	55 (68)	25 (31)	42(52) [2]	1 (2) [0, 1]
120 °C	+	5 min	60	31	26 (84)	5 (16)	29 (93) [2]	16 (55) [6, 10]
		10 min	60	39	29 (74)	10 (26)	28 (71) [2]	8 (28) [4, 4]
		>20 min	160	124	44 (35)	80 (65)	44 (35) [3]	0
150 °C	+	3 min	97	87	82 (94)	5 (6)	82 (94) [4]	51 (61) [10–16]
		>5 min	120	94	60 (64)	34 (36)	60 (64) [4]	0

PN: pseudo-pronucleus.

*All embryos were transferred into oviduct of recipient female.

## Discussion

In this study, we demonstrated that the nuclei of FD mouse spermatozoa have a strong tolerance not only to exposure to drying and vacuum treatment but also to frequent temperature changes or high and low temperatures.

The traditional definition of ‘tolerance’ is the recovery of cell membranes after exposure to extreme environments, and it is well known that mammalian cells or individuals cannot survive after being exposed to such conditions. However, recent advances in reproductive biotechnology allow us to resurrect mammalian species from ‘dead’ cell nuclei. For example, healthy offspring were obtained not only from frozen cadavers^7–9^, but also from FD mouse spermatozoa that had been preserved at RT for more than 1 year^14,15^ or even exposed to space radiation by preservation at the International Space Station for 9 months^18^.

Thus, the definition of life or tolerance should be reconsidered not only based on the survival of the body or its cells but also on the integrity of the cell nucleus. Using these new criteria, it is concluded that mammalian species, especially their spermatozoa, can also have a strong tolerance to extreme environments. In addition, it is not surprising that the spermatozoon has a strong tolerance to extreme environments because the transmission of correct genetic information to the next generation warrants high priority, to maintain the species permanently without mutation or degradation. This method of resurrection is artificial and requires reproductive biotechnology, but this tolerance of the nucleus offers great promise for the future of animal breeding. For example, the genetic diversity of mammalian species or genetically modified experimental animals can be maintained anywhere using FD spermatozoa, without the need for electric power, LN_2_ or expensive facilities.

In this study, the use of trehalose in the freeze-drying medium instead of glucose led to an increase in the tolerance of sperm nuclei. It is known that many tardigrades can survive under extreme dehydration conditions because of the accumulation of large amounts of trehalose in their bodies^26^. In addition, previous studies demonstrated that trehalose can protect spermatozoa from freezing or freeze-drying by acting as a cryoprotectant^24,25^. Therefore, it is likely that trehalose is an important factor to achieve tolerance to extreme environments in FD spermatozoa. However, recently, several tardigrade species were discovered that did not require trehalose for this tolerance^26,27^. In addition, usually, a cryoprotectant can protect the cell membrane or organelles; however, FD spermatozoa in this study were no longer viable. One possibility is that the increased tolerance of FD spermatozoa that was obtained using trehalose was achieved by just avoiding the Maillard reaction of glucose, rather than via the protectant effect of trehalose. In fact, previous studies demonstrated that FD spermatozoa can be preserved at room temperature for more than 1 year^15^ or at 40 °C for 1 month^28^ without trehalose. Additional experiments are required to understand the effect of trehalose.

In addition, our results might partially support or extend the Panspermia hypothesis^29,30^, which states that life could have been seeded on Earth via interplanetary objects. The major criticism of this hypothesis is that living organisms cannot survive long exposures to space radiation or high temperature^1^. Moreover, the hypothesis only proposes the transmission of ‘lower’ species, such as microorganisms; thus, it cannot explain the evolution of current life forms. However, our study suggests that at least the genetic information of spermatozoa might be maintained even after exposure to such extreme environments, and that it might be possible to convey the DNA or nuclei of ‘higher’ species through space, thereby spreading the sources of genetic information, which might then enable the evolution of more complex forms on Earth or similar planets.

## Materials and Methods

### Animals

BDF1 (C57BL/6N × DBA/2), BCF1 (C57BL/6N × C3H/He), C57BL/6N, C3H/He, DBA/2 and ICR mice (8–10 weeks of age) were obtained from SLC Inc. (Hamamatsu, Japan). Male mice of the 129B6F1 strain, which carry the gene encoding the green fluorescent protein (GFP) (GFPtg-129/Sv × GFP-tg-C57BL/6), were bred in our mouse facility; this was chosen as an example of a genetically modified mouse strain. Surrogate pseudo-pregnant ICR females, which were used as embryo recipients, were mated with vasectomized ICR males, the sterility of which had been demonstrated previously. On the day of the experiment or after finishing all experiments, mice were euthanized by CO_2_ inhalation or cervical dislocation and used for experiments. All animal experiments followed the Guide for the Care and Use of Laboratory Animals and were approved by the Institutional Committee of Laboratory Animal Experimentation of the University of Yamanashi.

### Preparation of FD spermatozoa

Both epididymides were collected from male mice, and ducts were cut using sharp scissors. A few drops of the dense sperm mass were then placed into a centrifuge tube containing 2 ml f HTF medium^31^ and incubated for 30 min at 37 °C in 5% CO_2_. In some experiments, spermatozoa were suspended in Tris-HCl^32^ with trehalose replacing glucose in the medium before freeze-drying. The concentration and activity of spermatozoa were measured, and 50 μl aliquots of the sperm suspension were dispensed into glass ampoules. In the preliminary experiments, we found that the sperm concentration did not affect the quality of FD spermatozoa; however, if sperm concentration and activity were too low, the sample was discarded, and sperm was collected again from another male. The ampoules were flash frozen in LN_2_ and freeze-dried using an FDU-2200 freeze-dryer (EYELA, Tokyo, Japan). The cork of the freeze-dryer was opened for at least 3 h until all samples were completely dry. After drying, the ampoules were sealed by melting the ampoule necks using a gas burner under vacuum, as described^18^.

### Treatment of FD spermatozoa with frequent temperature changes

The ampoules of FD spermatozoa were placed in a −30 °C freezer for 1 h, then taken out to RT (~25 °C) for 10 min. These ampoules were used as controls. Other experiments were repeated up to 10 times and ampoules were kept in a –30 °C freezer until use. In addition, some ampoules of spermatozoa from a BDF1 strain mouse were placed into LN_2_ for 10 min and taken out to the laboratory and kept for 10 min, which was repeated 10 times, similar to the freezer treatment described above. The control and experimental conditions were compared using ampoules derived from spermatozoa of the same individual.

### High-temperature treatment of FD spermatozoa

Before starting this study, we measured temperature changes in the oven that was used here before and after the door was opened using several thermometers. When the oven was set at 95 °C, the temperature fell to 93.5 °C immediately after the door was opened, but returned to 95 °C within 4 min of it being closed (Supplemental Fig. 1). The ampoules of FD spermatozoa were placed in the 95 °C oven for 30 min or up to 24 h. Then, ampoules were taken out and kept in a –30 °C freezer until use. For the 120 °C or 150 °C treatments, the temperature was set at least 1 day prior to the experiment. Ampoules were placed in the oven for the indicated duration and were then kept at −30 °C until use. For heat-treatment experiments, ampoules were exposed to a wide range of temperatures and periods; therefore, ampoules were derived from different individuals.

### Oocyte preparation

Female mice were super-ovulated via the injection of 5 IU of equine chorionic gonadotropin, followed by 5 IU of human chorionic gonadotropin (hCG) 48 h later. Cumulus–oocyte complexes (COCs) were collected from the oviducts of females 14–16 h later and moved to a Falcon dish containing HEPES-CZB medium^33^. To disperse the cumuli, COCs were transferred to a 50 μl droplet of HEPES-CZB medium containing 0.1% bovine testicular hyaluronidase for 3 min. Cumulus-free oocytes were washed twice and moved to 20 μl droplets of CZB medium^34^, for culture.

### Intracytoplasmic sperm injection (ICSI) and embryo transfer

ICSI was performed as described^33^. Just before starting ICSI, the neck of an ampoule was punctured and 50 µl of sterile distilled water was immediately added and mixed using a pipette. For microinjection of spermatozoa, 1–2 µl of the sperm suspension was moved directly to the injection chamber. The sperm suspension was replaced every 30 min during the ICSI procedure. Application of several piezo pulses separated the sperm head from the tail, and the head was then injected separately into the oocyte. The oocytes that survived ICSI were incubated in CZB medium at 37 °C under 5% CO_2_ in humidified air. Pronuclear formation was checked at 6 h after ICSI.

### Oocyte activation and embryo transfer

In some experiments, oocytes were activated after ICSI using 5 mM SrCl_2_ in Ca^2+^-free CZB medium for 1 h, followed by culture in CZB medium until embryo transfer. One day later, fertilized embryos that had reached the 2-cell stage were transferred into the oviducts of pseudo-pregnant ICR female mice at 0.5 days post coitum (dpc). These mice had been mated with a vasectomized male the night before transfer and were anaesthetized using a peritoneal injection of Avertin just before embryo transfer. At 19.5 dpc, the offspring were delivered by Caesarean section and randomly selected offspring were transferred to the cage of a foster mother who had delivered pups naturally.

### Observations of FD spermatozoa

The survival rates of FD spermatozoa were measured using live/dead assay kits (Molecular Probes, Thermo Fisher Scientific), according to the manufacturer’s instructions. For scanning electron microscopy observations, the samples were fixed with 2% formaldehyde, 2.5% glutaraldehyde in 0.1 M sodium cacodylate buffer (pH 7.4) for 2 h at room temperature. After washing with 0.1 M sodium cacodylate buffer (pH 7.4) three times, they were post-fixed in ice-cold 1% OsO_4_ in the same buffer for 2 h. The samples were then dehydrated in a graded ethanol series, transferred into isoamyl acetate and dried in a critical point drier (JCPD-5; JEOL, Tokyo, Japan) after substitution with liquid CO_2_. Dried samples were treated using an osmium coater (Neoc-STB, Meiwafosis Co. Ltd., Tokyo, Japan). Samples were examined under a scanning electron microscope (JSM 5600-LV, JEOL, Tokyo, Japan).

### Analysis and scoring of comet slides

The levels of DNA damage in irradiated spermatozoa were examined by comet assay, which can detect DNA damage in sperm nuclei. Sperm DNA damage, potentially caused by single- and double-stranded breaks^35^, was measured using Comet Assay® kits (Trevigen), according to the manufacturer’s instructions. Briefly, sperm specimens and their control (non-heated) counterparts were collected from two ampoules derived from the same individual immediately after opening and were rehydrated in water. Each specimen and its control counterpart were mounted on the same slide, and about 100 sperm heads on each slide were analysed after electrophoresis. To standardize the results across different irradiation treatments, the length of each DNA comet tail was divided by the mean length of the control tail in each experiment.

### Statistical analysis

The results of the comet DNA breakage assay were evaluated using the Wilcoxon–Mann–Whitney non-parametric test, the gamma-H2AX assay was evaluated using Student’s *t* tests, and the birth rates were evaluated using chi-squared tests. The statistical significance of any differences between variables was set at *P* < 0.05.

## Supplementary information

Extended Data and Figures
