# Supplementary material for: Tolerance of the freeze-dried mouse sperm nucleus to temperatures ranging from −196 °C to 150 °C
Source: Sci Rep. 2019 Apr 5;9:5719. doi: 10.1038/s41598-019-42062-8 (PMC6450870; doi:10.1038/s41598-019-42062-8)
Supplement: Supplementary file 1 — Extended Data and Figures [file 41598_2019_42062_MOESM1_ESM.pdf]

Extended Data and Figures

# Tolerance of the freeze-dried mouse sperm nucleus to temperatures ranging from –196 ° C to 150 ° C

Sayaka Wakayama, Daiyu Ito, Yuko Kamada, Shigenobu Yonemura, Masatoshi Ooga, Satoshi Kishigami, Teruhiko Wakayama

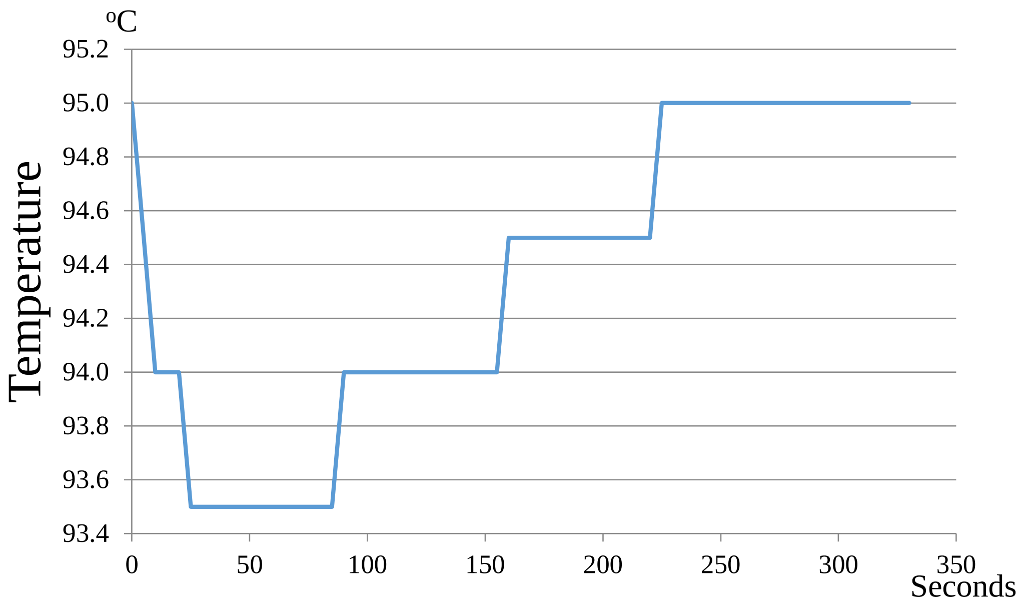


Supplemental Figure 1. Recovery time of oven temperature. The temperature of inside oven was measured each 5 seconds.

Supplemental Table 1. The rate of live or dead spermatozoa before and after freeze-drying

| Sperm condition | No. of examined spermatozoa | No. live sperm | No. dead sperm (%) |
|---|---|---|---|
| Fresh sperm | 132 | 121(92) | 11(8) |
| FD sperm | 80 | 0 | 80 (100) |

Supplemental Table 2. Observation of heat treated spermatozoa by SEM

| Sperm condition | Heat temp. ($^{o}$C) | No. examined sperm | Smooth surface (%) | Rough surface (%) | Raggedly surface (%) |
|---|---|---|---|---|---|
| Fresh sperm | RT | 10 | 10 (100) | 0 | 0 |
| | 95 | 18 | 0 | 4 (22) | 14 (78) |
| FD sperm | RT | 13 | 0 | 11 (85) | 2 (15) |
| | 95 | 28 | 0 | 15 (54) | 13 (46) |

Supplemental Table 3. Length of comet tail between control (non-heat) and heat treated FD spermatozoa with or without Treharose

| Treharose | Heat | No. of examined spermatozoa | Average length of comet tail | SD | P value Kruskal-Wallis test |
|---|---|---|---|---|---|
| - | - | 165 | 1.00 | 0.194 | - |
| - | + | 393 | 1.21 | 0.255 | < 0.05 |
| + | - | 167 | 1.00 | 0.189 | - |
| + | + | 265 | 1.01 | 0.173 | 0.715 |

Supplementary Table 4. Full term development of B6 mouse oocyte injected with freeze-dried B6 mouse spermatozoa treated with 95 °C for up to 2 h with oocyte activation and trehalose

| 95 °C heated time | No. injected oocytes | No. survived | No. (%) of oocytes 24 h after ICSI | | | No. of embryo transfer (Recip.) | No.(%) of offspring |
|---|---|---|---|---|---|---|---|
| | | | 2cell | 1cell | frag | | |
| 0 | 220 | 184 | 151(82) | 30(16) | 3(1) | 151(8) | 34(23) |
| 1h | 239 | 188 | 172(83) | 4(2) | 12(7) | 172(8) | 42(24) |
| 2h | 221 | 178 | 153(86) | 20(11) | 5(3) | 153(8) | 9(6) |
